# 24-hour movement behaviors in association with academic performance in children and adolescents: cross-sectional compositional data analysis

**DOI:** 10.1186/s44167-026-00099-x

**Published:** 2026-04-04

**Authors:** Heidi J. Syväoja, Tuomas Kukko, Piritta Asunta, Harto Hakonen, Janne Kulmala, Marja-Kristiina Lerkkanen, Pekka Räsänen, Tuija H. Tammelin

**Affiliations:** 1 Likes, School of Health and Social Studies, Jamk University of Applied Sciences, Piippukatu 2, 40100 Jyväskylä, Finland; 2 Department of Teacher Education, University of Jyväskylä, Jyväskylä, Finland; 3 Epilepsia Helsinki, Department of Pediatric Neurology, HUS Helsinki University Hospital, Helsinki, Finland; 4 Turku Research Institute for Learning Analytics, University of Turku, Turku, Finland

**Keywords:** Exercise, Sedentary behavior, Sleep, Reading, Mathematics

## Abstract

**Background:**

While physical activity (PA), sedentary time (ST), and sleep each show individual associations with learning outcomes, their combined associations remain largely unexplored. This cross-sectional study examined the association between the 24-h movement behavior composition and arithmetic and reading fluency in children and adolescents, gender differences in these associations, and theoretical 30‑minute behavior‑change scenarios.

**Methods:**

Volunteered children (N = 253, mean age: 9.5 ± 0.4 years, 53% girls) and adolescents (N = 174, mean age: 13.7 ± 0.6 years, 63% girls) participated. Children’s arithmetic fluency was measured using the FUNA test battery, and reading fluency with the Sentence Reading fluency test. Adolescents’ arithmetic fluency was measured with the Basic Arithmetic Test, and reading fluency with the word reading task ALLU. Light PA, moderate PA, vigorous PA, and ST were measured using hip-worn accelerometers (ActiGraph GT3X +). Students recorded their sleep time. The associations were examined using compositional data analysis. The ratios of behaviors were predictors within linear mixed models, adjusted for age, gender, special educational needs, body fat percentage, and guardians’ education.

**Results:**

The light PA relative to other behaviors was inversely associated with reading fluency (β = − 0.98,* p* = 0.014) among children. No other behaviors were associated with reading or arithmetic fluency in children or adolescents. Among adolescents, there was a significant interaction effect of gender and the time spent in ST on reading fluency (β = − 2,85, *p* = 0.037), but not on arithmetic fluency. Together, the interaction and main effects indicate that higher ST is linked to better reading fluency for both genders, with a stronger association in boys. Among adolescents, predicted 30‑minute reallocations from ST to sleep, or moderate PA, as well as from LPA to sleep, increased the difference in reading fluency between girls and boys.

**Conclusions:**

Lower levels of light-intense PA were linked to better children’s reading fluency. The association between the 24-h activity composition and reading fluency among adolescents may differ between boys and girls. From a 24-h movement behavior perspective, supporting reading may require strategies that are tailored by age and gender.

**Supplementary Information:**

The online version contains supplementary material available at 10.1186/s44167-026-00099-x.

## Background

Physical activity (PA), sedentary time (ST), and sleep form a 24-h movement behavior composition, as they occur within a finite period, and the time spent on one behavior inevitably replaces the time spent on others within the same 24 h [1, 2]. Each of these movement behaviors has been associated with academic performance, which is built on basic reading and math skills [3, 4]. Higher levels of PA, especially moderate-to-vigorous PA (MVPA), have been associated with better academic performance [5], likely through cognitive and motivational benefits such as brain structural and functional adaptations, increased engagement, and improved self-esteem [6–8]. The association between ST and academic performance has been more inconsistent [9, 10]. The content of sedentary activities may play a role: cognitively engaging activities, such as reading or educational gaming, may foster learning, whereas passive behaviors, like watching TV or scrolling social media, can have detrimental effects [11]. However, excessive ST may also indirectly impair academic outcomes through mechanisms such as reduced aerobic fitness [12]. Sleep has been positively associated with academic performance, with proposed mechanisms including memory consolidation, reduced daytime sleepiness, and enhanced alertness and cognition [13].

Evaluating these movement behaviors together has been proposed as important for promoting and maintaining overall well-being [14]. Compositional data analysis (CoDA) has been utilized to measure the associations between 24-h movement behavior composition and physical [15–18], mental [19–22], psychosocial [21–24], and cognitive health [19, 22, 25, 26] in children and adolescents. Although academic performance is closely related to overall well-being [27], the combined influence of movement behaviors on academic achievement has received little attention [28, 29]. A systematic review by Wilhite et al. [28] found that only a few studies investigated the associations between combinations of movement behaviors and academic performance, with just one considering all three behaviors together, and none applying CoDA. The use of CoDa is recommended because it analyzes the trade-offs in movement behaviors while accounting for dose–response associations [2, 28].

A couple of recent studies have utilized CoDa to explore the associations of 24 h-movement behavior with academic performance: Watson et al. [30] showed that lower levels of light PA (LPA) were associated with better numeracy and literacy, while higher ST was linked to better literacy relative to time spent in other movement domains among children aged 10–12. According to Padmapriya et al. [31], the composition of 24-h movement behaviors at ages 5.5 and 8 years was not associated with academic achievement; however, higher MVPA at age 5.5 relative to other behaviors predicted lower academic achievement at age 9, while reallocating MVPA to sleep was linked to higher scores. Dumuid et al. [32] aimed to identify an optimal time-use composition for health and well-being among 11–12-year-old children. They found that days with the highest ST and lowest PA were optimal for cognitive and academic outcomes, whereas the optimal scenario for overall health and well-being comprised 10.4 h of sleep, 9.7 h of ST, 2.4 h of LPA, and 1.5 h of MVPA. According to a systematic review and meta-analysis by Kuzik et al. [29], among children aged 8–16, most 24-h movement behaviors showed no association with academic or cognitive outcomes, whereas LPA was generally inversely associated with these outcomes. Similarly, Bourke et al. [22], in their systematic review and compositional meta-analysis, found no significant associations between 24-h movement behavior compositions and cognitive or academic outcomes in children and adolescents aged 3–18 years. Although non‑significant, they observed a trend suggesting that more ST and less LPA might relate to better cognitive functions [22]. The authors of these studies [29–32] emphasized the importance of promoting sufficient MVPA and sleep, reducing excessive ST, and suggested that LPA may displace time from other movement behaviors that are more beneficial for academic achievement.

Using CoDA analysis, the relationship between movement behaviors and academic achievement has been studied less among adolescents older than 12 years [29]. Transition from early childhood to adolescence alters the time spent on various movement behaviors [33, 34], with distinct patterns emerging between boys and girls [35]. This may also modify the relationship between a 24-h movement behavior composition and academic performance.

Given the limited research in this area, further studies are needed to examine the combined association of PA, ST, and sleep with academic performance in childhood and adolescence. Schools play an important role in enhancing learning and supporting appropriate composition of the movement behaviors that promote health and well-being, especially in enhancing PA and reducing ST [36]. Understanding how these behaviors interact and influence academic outcomes is essential for educators, parents, and policymakers to develop environments and practices that support learning and well-being both during school and leisure time. Therefore, using the CoDA approach, we studied the association of the 24-h movement behavior composition (sleep, ST, LPA, moderate PA [MPA], and vigorous PA [VPA]) with basic math and reading skills (arithmetic and reading fluency) in children and adolescents, assessed potential gender modification, and additionally evaluated theoretical 30‑minute behavior‑change scenarios.

## Methods

### Study design and participants

**Fig. 1:**
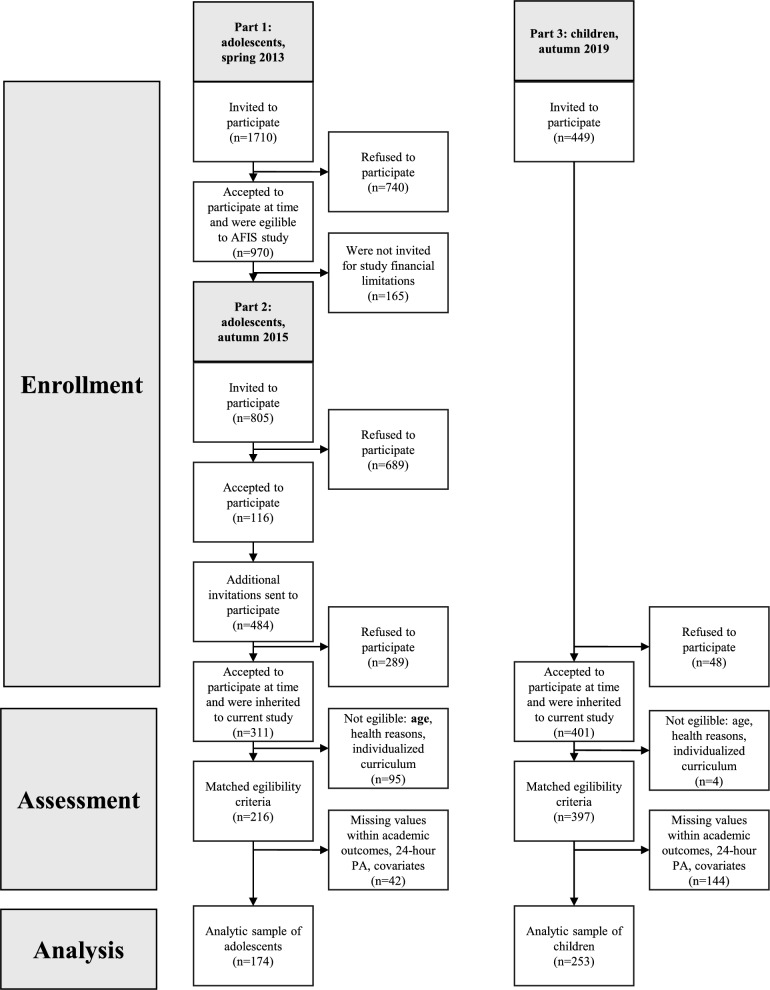
A flow diagram of the study design

This cross-sectional study utilized baseline data from two completed Finnish studies: the 5-month Moving Maths multi-arm cluster randomized controlled trial (N = 397, mean age: 9.5 ± 0.4 years, 50% females) (ISRCTN71844310, registered 10 April 2019, https://doi. org/10. 1186/ISRCT N7184 4310) [37] and the finished Active, Fit and Smart study (AFIS) (N = 311, mean age: 14.0 ± 1.1 years, 59% females) [38]. Participants for the Moving Maths study were recruited in autumn 2019 from 13 eligible schools in Central Finland. Altogether, 397 volunteered third-grade students formed the study population of the Moving Maths study and represents children in this study [37]. Participants for the AFIS study were recruited in spring 2015 from five schools representing a broad geographical area of Finland, and in autumn 2015 from two schools in Central Finland. A total of 311 eligible 6th–9th grade students volunteered to participate [38], of which seventh and eighth graders were selected to represent the adolescents in this study. The flow diagram of the study design is presented in Fig. 1. In both studies, students and their guardians signed an informed consent to participate. Both studies were performed according to the principles of the Declaration of Helsinki and were approved by the Ethics Committee of the University of Jyväskylä.

### Sample size estimation and justification

The sample size of the children’s data set was initially planned for detecting medium main and moderating effects between groups and specific covariates within cluster-randomized controlled trials with pre- and post-intervention measurements [37]. Adolescents’ data set was initially collected for path models with multiple nodes, including mediating effects, for which the sample size was slightly small [38]. The adequacy of the two samples for the purposes of this study was illustrated by critical effect size analysis. In the analysis, we calculated the cutoff values for increment for marginal R-squared that would be detected with a significance level alpha of 0.05, statistical power of 80 percent, and the observed sizes of analytic samples (children’s n = 253, adolescents’ n = 174). The critical effect sizes were ΔR2 = 0.035 for children and ΔR2 = 0.050 for adolescents.

### Assessment of academic performance

Reading and math skills were assessed using standard group-administered tests at the schools by trained research assistants. The academic performance within the age groups, children and adolescents, was assessed by different age-appropriate methods.

*Arithmetic fluency:* Arithmetic fluency in children was assessed with an online FUNA (Functional Numeracy Assessment Dyscalculia Battery) test battery [39], which includes arithmetic reasoning, single-digit addition, single-digit subtraction and multi-digit addition and subtraction tasks. In the arithmetic reasoning task, the children had to find the arithmetic logic to continue the four numbers within a 4-min time limit. In the single-digit addition and subtraction tasks, the children had to solve tasks were the addends or the differences were between 1 and 9 within the 2-min time limit. In the multi-digit calculation task, 2- to 3-digit numbers were mixed, and children had to solve as many addition and subtraction tasks as possible within the 3-min time limit. In all the tasks, arithmetic performance was calculated by the total number of correct answers and standardized with reference data from a Finnish third-grade normative sample (n > 18,000) [40]. The average of the z-scores of these tasks indicated arithmetic fluency.

The Basic Arithmetic Test, a pen-and-paper test, was used to determine the arithmetic fluency of adolescents [41]. In this test, students were asked to perform as many addition, subtraction, multiplication, and division tasks as possible within a 3-min. Arithmetic performance was calculated based on the number of correct answers and standardized with reference data from the Finnish First Steps study [42].

*Reading fluency:* Reading fluency in children was assessed with an online Sentence Reading fluency test, a Finnish adaptation of the Woodcock-Johnson reading fluency task with time limit [43]. In this test, children were presented with sentences and had to choose whether the argument was true or false. For example, “blueberries are green”. They had 90 s to answer as many problems as possible. Reading fluency was calculated by the number of correct answers and was standardized with reference data from the Finnish normative sample (n > 18,000) of the FUNA test battery [40].

Adolescent reading fluency was assessed with a pen and paper test called ALLU—Ala-asteen lukutesti (ALLU—Reading test for lower primary school) [44]. In this test, each of the 80 items consists of a picture with four phonologically similar words attached to it, and students were asked to silently read the four words and then draw a line to connect the picture with the right word, semantically matching it. For example, there is a picture of a bunny (in Finnish, pupu) and the correct word, along with three distractors (English word is in parentheses): pipo (cap), papu (bean), and apu (help). Reading fluency was calculated by the number of correct answers within a 2-min time limit. The counts of correct answers were standardized to the z-scores within the two school grades (7th/8th grade).

### Assessment of 24‑h movement behavior composition

PA and ST were monitored with GT3X + and wGT3X + accelerometers (ActiGraph, Pensacola, FL). Data collection was carried out using widely used methods and recommendations [45]. Students wore the monitor on their right hip during waking hours for seven full consecutive days, excluding water-based activities. Data were collected in raw 30-Hz acceleration, underwent standard filtration and converted into 15-s epoch counts. A customized Visual Basic macro for Excel software was used for data reduction. For children, at least 600 min per day measured data on four days or more were needed for a valid monitoring period. For adolescents, at least 500 min per day of measured data on four days or more were required for a valid monitoring period. For both data, periods of 30 min of consecutive zero counts were defined as non-wear times and spurious accelerations (> 20,000 counts per minute [CPM]) were ruled out. The Evenson’s cut points, validated among children and youth [46, 47], were utilized to calculate ST (≤ 100 CPM), LPA (≥ 101 counts per minute [CPM]), MPA (≥ 2296 CPM), and VPA (≥ 4012 CPM).

Since the accelerometer was not worn during the water activities and cannot reliably account for all activities, such as cycling, the time spent in swimming and cycling was assessed using diaries filled out by the students at the same time as the accelerometer measurement. Students reported their swimming and cycling time with an accuracy of 10 min daily. Swimming and cycling correspond to an intensity of a minimum of 3.5 metabolic equivalents [48], and the time spent in these activities was added into MPA. The daily average swimming times from diaries were added without any reductions to the device-measured time. For cycling, the accelerometers (mounted on hip) were assumed to underestimate the intensity of PA [49] and therefore, the daily average times spent cycling from the diaries were added into MPA and subtracted from LPA. The sleep time was also assessed using the same diary. Students reported their wake-up and bedtime times, and the sleep time was calculated by taking into account the next day’s wake-up time and the previous day’s bedtime (sleep time from six nights).

### Assessments of potential confounders

According to previous literature [5], age, gender, special educational needs, body fat percentage, and guardians’ education have been identified as confounding factors. Children’s special educational needs were assessed using a questionnaire completed by teachers. During the period of data collection, Finnish basic education implemented a three-tiered support model consisting of general, intensified, and special support to ensure equitable and adequate educational assistance for all students. Teachers marked if the child was offered intensified support (regular support for their learning or school attendance) or special support (if growth, development, and learning objectives are not sufficiently met with other supportive measures) or no support (1 = intensified or special support, 0 = no support) [50]. Adolescents’ special educational needs were assessed with an online questionnaire completed by a parent or guardian. They answered the question: “Does your child have any diagnosed learning difficulties?” (categorization: 1 = yes and 0 = no or do not know). Body fat percentage was measured using an InBody 720 analyzer (InBody, Seoul, South Korea). In the online questionnaire, each participant’s parent or guardian reported the guardian’s educational level, from which the highest level of guardians’ education was calculated (categorization: 1 = tertiary level education and 0 = basic or upper secondary education).

### Statistical analyses

Descriptive statistics were calculated for adolescents’ and children’s samples separately and combined across the samples. These were percentages for categorical variables, means and standard deviations for continuous variables, and geometric means for behavioral composition variables. The differences between samples were evaluated by chi-squared and* t*-tests, where applicable. In addition, the characteristics of the analytic sample (completely observed) participants were compared to those of the excluded ones.

There were no zero values within the five behavior variables (sleep, ST, LPA, MPA, and VPA). The mean compositional ratios and variation matrices were calculated. The differences between the two samples were visualized, with a detailed description provided in the supplementary material. The dependent behavior composition was transformed into four linearly independent predictors by isometric log-ratio (IRL) transformations [2, 51]. The behavior pattern was rotated five times, such that each component at its turn was contrasted to the geometric mean of the other behaviors in the first ratio.

The association between academic performance and behavioral compositions was analyzed using linear mixed-effects models. All analyses were replicated for the two outcomes: arithmetic and reading fluency, and stratified by age categories: children and adolescents. All models were adjusted for age (centered continuous decimal age), gender (0 = boy, 1 = girl), special educational needs (0 = no, 1 = yes), body fat percentage (centered continuous proportion), and guardians’ education (0 = lower education level, 1 = tertiary education level). The rotated ILR coordinates were added as fixed effects to the models. School classes were set to have random intercepts to confront the clustered data structure.

Model performance was evaluated by calculating marginal R-squared, intra-class correlation coefficients (ICC), and root mean squared error (RMSE). Marginal R-squared gives the proportion of variance explained by the fixed effects, ICC reflects the relative variation of the random intercepts, and RMSE quantifies the typical value of residual errors.

Explanatory power of behavior composition was assessed by comparing the fitted models with a more restricted model. The more compact model included only fixed effects for adjusting factors and the random intercepts. The Wald statistics and their significance were calculated to determine if the ILR coordinate pattern had a multivariate association with the outcomes. Also, the increment in marginal R-squared was calculated to quantify the potentially achieved better predictability.

To test if gender moderated the association of academic performance outcomes and behavior composition, the interaction terms for gender and ILR coordinates were included in each model. The model performance indices were calculated for the models of main effects. In the calculation of explanatory power, the interaction model was compared to the corresponding model of main effects only.

The final phase of analysis was performed to predict the effects of isotemporal behavior-change scenarios. In each scenario, 30 min of daily behavior was reallocated between the 24-h movement behavior composition components. The reallocation effect was calculated to interpret the statistically significant associations. Therefore, the predictions were made from the model of main effects for the children’s sample and from the model with interaction terms for adolescents. Effects were visualized by calculating and plotting the predicted differences from sample means and confidence intervals for a given reallocated time usage.

All analyses were performed in R (version 4.2.4) using the following external packages: compositions (CoDA procedures), lme4 (linear mized effects models and bootstrapping the predictions of scenarios), lmerTest (Wald tests for single parameters), performance (model performance and coefficients of determination), simr (critical effect size calculations) and WeMix (Wald tests for sets of ILR parameters).

## Results

### Descriptive statistics

Two hundred fifty-three children (9.5 ± 0.4 years, 53% girls) and 174 adolescents (13.7 ± 0.6 years, 63% girls) provided valid accelerometer data and complete information on arithmetic and reading fluency and potential confounders (age, gender, special educational needs, body fat percentage, guardians’ education), forming the total analytic sample for this study. Sample-specific distributions and sample differences are presented in Table 1. Children spent more time sleeping, LPA, MPA, and VPA than adolescents, while adolescents had more ST (Table 1). Compared with the excluded sample, child analytic participants showed higher reading fluency and fewer special educational needs, whereas adolescents showed higher LPA and MPA; and guardians were more educated in both analytic samples (Fig. 1, Table S1).

**Table 1:** Descriptive statistics of samples and sample differences

Variables	All n = 427	Moving maths sample, children(ages 9‒11) n = 253	AFIS sample, adolescents (ages 12‒15) n = 174	Difference *p* value
Categorical variables (%)				
*Gender (%)*				
Girls	57.1	53.0	63.2	0.045*
Boys	42.9	47.0	36.8	
*Special educational needs (%)*				
No	91.1	89.7	93.1	0.302
Yes	8.9	10.3	6.9	
*Guardians’ education (%)*				
Tertiary education level	75.4	77.5	72.4	0.281
Lower than tertiary level	24.6	22.5	27.6	
*Continuous variables: M (SD)*				
Age (years)	11.2 (2.1)	9.5 (0.4)	13.7 (0.6)	< 0.001**
Body fat (%)	18.3 (8.4)	17.7 (8.5)	19.2 (8.1)	< 0.001**
Arithmetic fluency (std score)	0.00 (1.00)	0.00 (1.00)	0.00 (1.00)	1.000
Reading fluency (std score)	0.00 (1.00)	0.00 (1.00) ^1^	0.00 (1.00)	1.000
*Daily average movement behavior*				
Sleep (h/d)	9.7 (0.8)	10.1 (0.5)	9.2 (0.7)	< 0.001**
Sedentary time (h/d)	7.8 (1.3)	7.0 (0.9)	8.9 (1.1)	< 0.001**
Light PA (h/d)	3.7 (1.0)	4.2 (0.8)	3.0 (0.7)	< 0.001**
Moderate PA (min/day)	62.1 (26.2)	68.5 (24.1)	52.8 (26.5)	< 0.001**
Vigorous PA (min/day)	21.7 (11.7)	23.1 (11.6)	19.7 (11.7)	0.004**
Accelerometer wear time (h/d)	12.9 (0.7)	12.8 (0.7)	13.1 (0.8)	< 0.001**
Recorded time in total (h/d)^2^	22.7 (0.9)	22.9 (0.6)	22.3 (1.0)	< 0.001**
Number of valid days	6.1 (1.0)	5.9 (1.0)	6.4 (0.9)	< 0.001**
Number of valid nights	5.8 (0.6)	5.8 (0.7)	5.9 (0.5)	0.241
*Geometric means of behavior composition*				
Sleep (%)	42.7	44.0	41.0	
Sedentary time (%)	34.0	30.5	39.7	
Light PA (%)	16.0	18.3	13.2	
Moderate PA (%)	4.2	4.7	3.5	
Vigorous PA (%)	1.4	1.5	1.2	

For categorical variables, percentages are provided for the overall sample and by age group. For continuous variables, means and standard deviations are presented. The differences in proportions are tested using the chi-squared test, and the differences in means are tested using Student’s* t*-test

M, mean; SD, standard deviation; PA, physical activity; * *p* < 0.05, ** *p* < 0.01

1 Standardization by population level reference values gave: M (SD) of 0.062 (1.041). However, all z-scores are re-standardized here within the age group to ensure a comparable basis for further analysis

2 Includes accelerometer measurements and completing information from diaries (sleep, cycling, and swimming)

The 24‑h movement behavior composition summaries are presented in supplementary material (Tables S2, S3, and Fig. S1). Table S2 presents the mean ratios of parts of 24-h movement behavior composition for the full sample and both age groups. For example, the sleep time is, on average, 1.3 times greater than the ST, 2.7 times greater than the LPA, 10.3 times greater than the MPA and 31.3 times greater than the VPA. Within the variation matrix in Table S3, the behaviors with common values close to zero varied with high codependency between individuals, and behaviors with higher values indicated divergent ratios between individuals. Figure S1 presents the empirical density function estimates of the parts of the 24-h movement behavior composition.

In the supplementary material (Fig. S2), the 24-h movement behavior composition (relative behavioral profiles) for children and adolescents grouped by arithmetic or reading fluency tertiles is presented. The relative distribution of times for each group is presented as the log ratio between the group compositional mean and the overall compositional mean times spent in sleep, ST, LPA, MPA, and VPA.

### Cross‑sectional associations of 24-hour movement behavior composition with academic performance

Table 2 presents the regression models for the associations of 24-h movement behavior composition and academic performance in both study samples. Among children, the time spent in LPA relative to other behaviors was inversely associated with reading fluency (β = − 0.98, *p* = 0.014). Any other 24-h movement behaviors relative to each other were not associated with reading or arithmetic fluency (Table 2). Among adolescents, none of the 24-h movement behaviors relative to each other were associated with arithmetic or reading fluency (Table 2).

**Table 2:** Fixed effects of linear mixed models for arithmetic and reading fluency by age groups

Outcome	Explanatory variable	Children (ages 9‒11), n = 253 in 22 groups				Adolescents (ages 12‒15), n = 174 in 34 groups			
		Beta	SE (Beta)	95% CI	*p *value	Beta	SE (Beta)	95% CI	*p* value
Arithmetic fluency									
	Age (years)	0.25	0.17	− 0.07, 0.58	0.134	− 0.01	0.14	− 0.28, 0.27	0.971
	Gender (girl)	− 0.24	0.13	− 0.49, 0.00	0.056	− 0.04	0.19	− 0.40, 0.32	0.829
	Special educational needs (yes)	− **0.98**	**0.20**	− **1.36, −0.59**	< 0.001**	− 0.52	0.30	− 1.10, 0.05	0.084
	Body fat (%)	− 1.28	0.78	− 2.86, 0.24	0.104	− 1.92	1.10	− 4.02, 0.19	0.083
	Guardians’ education (tertiary education)	0.20	0.14	− 0.07, 0.48	0.151	0.25	0.17	− 0.08, 0.58	0.145
	*First ILR coordinate of rotated behavior composition*								
	Sleep vs. others	− 1.30	0.72	− 2.68, 0.08	0.071	− 1.19	0.84	− 2.80, 0.42	0.160
	Sedentary time vs. others	0.58	0.45	− 0.29, 1.46	0.200	0.76	0.66	− 0.50, 2.02	0.250
	Light PA vs. others	0.41	0.38	− 0.31, 1.14	0.273	0.10	0.35	− 0.57, 0.77	0.776
	Moderate PA vs. others	0.15	0.21	− 0.25, 0.56	0.460	0.14	0.20	− 0.25, 0.52	0.500
	Vigorous PA vs. others	0.15	0.14	− 0.13, 0.43	0.297	0.19	0.15	− 0.10, 0.48	0.209
	Model performance indices	ICC = 0.104	RMSE = 0.851	R^2^ = 0.144		ICC = 0.000^1^	RMSE = 0.954	R^2^ = 0.081	
	Explanatory power of behavior composition	Wald = 4.67	p = 0.323	ΔR^2^ = 0.012		Wald = 4.35	p = 0.361	ΔR^2^ = 0.018	
Reading fluency									
	Age (years)	0.03	0.18	− 0.32, 0.37	0.872	0.03	0.15	− 0.25, 0.31	0.816
	Gender (girl)	0.17	0.13	− 0.09, 0.43	0.207	**0.59**	**0.18**	**0.24, 0.95**	0.002**
	Special educational needs (yes)	− **0.47**	**0.21**	− **0.87, −0.05**	0.026*	− 0.57	0.30	− 1.13, 0.01	0.056
	Body fat (%)	− 0.04	0.83	− 1.68, 1.56	0.962	− **2.19**	**1.07**	− **4.24, −0.12**	0.043*
	Guardians’ education (tertiary education)	0.19	0.15	− 0.10, 0.48	0.207	0.28	0.17	− 0.04, 0.61	0.098
	*First ILR coordinate of rotated behavior composition*								
	Sleep vs. others	1.42	0.76	− 0.06, 2.88	0.064	− 0.67	0.83	− 2.24, 0.93	0.416
	Sedentary time vs. others	− 0.30	0.48	− 1.22, 0.64	0.535	0.31	0.65	− 0.95, 1.54	0.636
	Light PA vs. others	− **0.98**	**0.40**	− **1.74, −0.21**	0.014*	0.29	0.35	− 0.38, 0.95	0.409
	Moderate PA vs. others	− 0.15	0.22	− 0.58, 0.28	0.486	− 0.00	0.20	− 0.39, 0.37	0.993
	Vigorous PA vs. others	0.01	0.15	− 0.28, 0.30	0.933	0.08	0.15	− 0.20, 0.37	0.584
	Model performance indices	ICC = 0.066	RMSE = 0.918	R^2^ = 0.064		ICC = 0.023	RMSE = 0.919	R^2^ = 0.109	
	Explanatory power of behavior composition	Wald = 12.35	*p* = 0.015*	ΔR^2^ = 0.024		Wald = 1.96	p = 0.742	ΔR^2^ = 0.007	

1 Variance of random effects is estimated close to zero

PA, physical activity; *p < 0.05, **p < 0.01

The columns represent unstandardized regression coefficients (Beta) and their standard errors (SE), 95 percent confidence interval (CI), and significance approximated by Satterthwaite’s degrees of freedom (*p*-value). For each model, there are model performance indices: intra-class correlation coefficient (ICC), marginal coefficient of determination for all fixed effects (R^2^), and root mean squared error (RMSE). The explanatory power of behavior composition is quantified by Wald’s test statistic for comparison of the model with PA predictors and covariates only (Wald), p-value related to Wald test statistic (p), and increase of the coefficient of determination for IRL-coordinates (ΔR^2^). Statistically significant values are shown in bold

ICC values for random intercepts varied between 0.000 to 0.104 indicating none to little within-group correlations (Table 2). The marginal R-squared values for the models varied between 0.064 and 0.144. ILR coordinates increased R-squared values from 0.007 to 0.024 units, and the highest addition was observed in the children’s model of reading fluency (Table 2). Children’s model of reading fluency also indicated significant improvement compared to the model without ILR coordinates (Wald statistic 12.35, *p* = 0.015).

### Gender as a modifier of the association between 24-hour movement behavior composition and academic performance

Gender and the five 24-h movement behaviors did not have statistically significant interaction effects on arithmetic or reading fluency among children (Table S4). However, among adolescents, there was a significant interaction effect of gender and the time spent in ST on reading fluency (β = − 2,85, *p* = 0.037), but not on arithmetic fluency (Tables 3, S4). Interpreting the significant interaction and the main effects together suggests that higher levels of ST are associated with better reading fluency in both boys and girls, with a stronger association observed among boys. Gender and other movement behaviors did not have statistically significant interaction effects on arithmetic or reading fluency among adolescents (Table 3). Moderator model for adolescents’ reading fluency indicated an addition of 0.036 units to the R-squared. Wald test statistic (12.78, *p* = 0.012) suggested a significant improvement compared to the model of main effects only (Table 3).

**Table 3:** The fixed effects of the linear mixed effects model with gender-moderation for reading fluency in adolescents

Explanatory variable	Beta	SE (Beta)	95% CI	*p* value
Gender (girl)	− 0.07	1.22	− 2.38, 2.23	0.957
Special educational needs (yes)	− 0.59	0.30	− 1.15, −0.02	0.047
Guardians’ education (tertiary education)	**0.34**	**0.17**	**0.02, 0.66**	0.048*
Age (years)	0.02	0.15	− 0.25, 0.30	0.879
Body fat (%)	− 1.62	1.11	− 3.72, 0.50	0.148
*First ILR coordinate of rotated behavior composition*				
Sleep vs. others	− **6.05**	**3.05**	− **11.82, −0.28**	0.049*
Sedentary time vs. others	**4.75**	**2.26**	**0.43, 9.03**	0.038*
Light PA vs. others	1.59	1.25	− 0.75, 3.97	0.202
Moderate PA vs. others	− 0.77	0.77	− 2.23, 0.69	0.320
Vigorous PA vs. others	0.47	0.52	− 0.51, 1.45	0.361
*Interaction terms of gender and first IRL-coordinate*				
Sleep vs. others	3.37	1.77	0.03, 6.72	0.059
Sedentary time vs. others	− **2.85**	**1.36**	− **5.42, −0.27**	0.037*
Light PA vs. others	− 0.74	0.72	− 2.13, 0.61	0.304
Moderate PA vs. others	0.46	0.43	− 0.36, 1.28	0.288
Vigorous PA vs. others	− 0.23	0.30	− 0.80, 0.34	0.442
Model performance indices	ICC = 0.028	RMSE = 0.894	R^2^ = 0.145	
Explanatory power of behavior composition	Wald = 12.78	*p* = 0.012*	ΔR^2^ = 0.036	

PA, physical activity

*p < 0.05, **p < 0.01.

Interaction terms for gender and the four ratios of the 24‑h behavioral composition are included into the model

The columns represent the unstandardized regression coefficient (Beta) and its standard error (SE), 95 percent confidence interval (CI), and significance approximated by Satterthwaite’s degrees of freedom (*p*-value)

There is also a single value of intra-class correlation coefficient (ICC), marginal coefficient of determination for fixed effects (R2), and root mean squared error (RMSE)

The explanatory power of interaction terms of gender and behavior composition is quantified by Wald’s test statistic for comparison of the model with interaction terms and main effects only (Wald),* p*-value related to Wald test statistic with df = 4 (p), and increase of the coefficient of determination for interaction terms (ΔR2). Statistically significant values are shown in bold

### Theoretical behavior-change scenarios in arithmetic and reading fluency

**Fig. 2:**
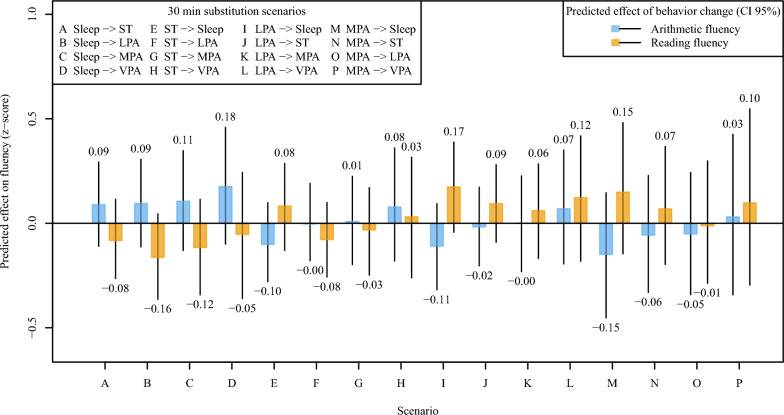
Predicted effects of behavior-change scenarios on arithmetic and reading fluency in children. Predicted effects of isotemporal substitution scenarios made to average behavior composition on arithmetic (blue) and reading (orange) fluency are visualized by colored bars, and the 95 percent confidence intervals (CI 95%) of the predictions by solid black lines. A statistically significant effect would be indicated by the corresponding confidence interval not crossing the horizontal line at zero. The amount of VPA was not sufficient for 30-min substitutions

Figure 2 presents the predicted effects of behavior-change scenarios in arithmetic and reading fluency in children. The figure includes 30‑minute isotemporal substitutions between behaviors within the 24‑hour composition. None of the predictions, assuming 30-min isotemporal substitutions, differed statistically significantly from the overall average among children.

**Fig. 3:**
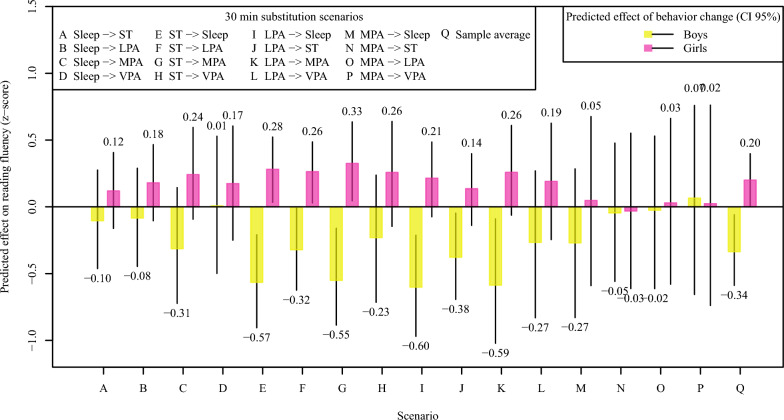
Predicted effects of behavior-change scenarios on reading fluency in adolescent boys and girls. Predicted effects of isotemporal substitution scenarios made to average behavior composition on reading fluency for adolescent boys (yellow) and girls (pink) are visualized by colored bars, and the 95 percent confidence intervals (CI 95%) of the predictions by solid black lines. A statistically significant effect would be indicated by the corresponding confidence interval not crossing the horizontal line at zero. The amount of VPA was not sufficient for 30-min substitutions

Figure 3 presents the predicted effects of behavior-change scenarios in reading fluency in adolescent boys and girls separately. The 30-min substitutions are made within the constrained 24-h behavioral composition. Considering the changes both in the prediction means and their confidence intervals, the 30-min reallocations from ST to sleep, or MPA, as well as from LPA to sleep, appear to widen the difference in reading fluency between boys and girls, suggesting improved reading fluency among girls after ST reallocations, but reduced fluency among boys after reallocations of both ST and LPA.

## Discussion

### The main findings of the study

This study examined the association of the 24-h movement behavior composition with basic math and reading skills (arithmetic and reading fluency) in children and adolescents, assessed whether these associations are modified by gender, and tested theoretical 30‑minute behavior‑change scenarios. The study results showed that higher amounts of LPA were inversely associated with reading fluency in children. No further behaviors were related to reading or arithmetic fluency in children or adolescents. Among adolescents, gender significantly moderated the association between ST and reading fluency, with higher ST relating to better reading fluency in both genders but more strongly in boys. Furthermore, predicted 30‑minute reallocation from ST to sleep, or MPA, as well as from LPA to sleep, increased the gender difference in reading fluency.

### The association of 24-hour movement behavior composition with academic performance among children

To our knowledge, this is one of the first studies to show the association of combinations of five movement behaviors (sleep, LPA, MPA, VPA, ST) with academic performance in children [28, 29]. Sleep, MPA, VPA, or ST were not associated with reading fluency. However, lower levels of LPA were associated with better reading fluency, which is in line with previous studies [29, 30]. Watson et al. [30] speculated that poorer academic achievement is unlikely to be related to higher amounts of LPA per se. Instead, the inverse association may arise because spending more time in LPA reduces the time available for other behaviors that support learning. Increasing LPA necessarily displaces time from sleep, MVPA, and sedentary study activities, behaviors known to benefit academic performance [30]. Literacy levels have declined in many countries, including Finland [52], and reduced reading exposure may contribute to weaker reading fluency. When reading fluency is weaker, reading tasks are less efficiently automatized and therefore demand greater attentional resources and executive control [53]. For example, reading skills improve through reading, which requires calm concentration and remaining still, resulting in more ST. Sufficient sleep and MVPA may enhance attention and executive functions [13, 54] and thereby improve children’s ability to concentrate on reading.

To explore this interpretation further, we theoretically tested these potential time reallocations in supplementary CoDA analyses. In these analyses, we modeled how changes in the 24‑h movement composition, for example, reallocating 30 min of LPA to MPA, might influence academic outcomes. However, none of the theoretical behavior-change scenarios were associated with children’s reading fluency. Nevertheless, when considered alongside the above-mentioned evidence, it could be suggested that ensuring sufficient sleep, adequate MVPA, and regular reading exposure, while reducing LPA, may help support reading fluency in children.

Contrary to the findings of Watson et al. [30], any 24-h movement behavior was not associated with arithmetic fluency. Singh et al. [55] in their meta-analysis concluded that there is strong evidence for the beneficial effects of PA on math performance. This beneficial effect is thought to result from PA’s positive impact on brain functions that support attentional resource allocation, conflict monitoring, cognitive preparation and processing speed during tasks that demand higher levels of executive control [5, 6]. Mathematics tasks can be divided into three levels based on their cognitive demands: knowing, applying, and reasoning [4]. Tasks at the applying and reasoning levels require higher levels of attention and executive function. However, in the present study we assessed only basic arithmetic skills, which may partly explain why PA, regardless of intensity, was not statistically significantly associated with arithmetic fluency.

In previous studies examining the association between MVPA and academic performance but not concerning the MVPA as part of the 24-h composition, self-reported MVPA has been positively associated with academic performance. In contrast, accelerometer-measured MVPA has not had the same systematic association with academic performance [56]. The inconsistency between self-reported and accelerometer-measured MVPA has been speculated to be due to children’s difficulties in evaluating their PA relative to accelerometers or accelerometers’ inability to measure cycling, swimming or other activities that hardly accumulates activity counts (e.g., skill-specific activities including agility, balance, control and coordination) [56]. Better motor skills have been shown to predict better academic performance [57], and skill-specific activities, including coordinative and cognitively engaging PA, may enhance cognitive and learning outcomes [58]. In this study, we sought to address the limitations of accelerometers for measuring coordinative PA and included the time spent in swimming and cycling, estimated by children themselves, in MPA.

### The association of 24-hour movement behavior composition with academic performance among adolescents

In this study, none of the five 24-h movement behaviors, relative to others, showed a significant association with academic outcomes among adolescents. The result is somewhat inconsistent with our findings concerning children in this as well as with previous findings in younger samples [29, 30]. To our knowledge, this is the first study to assess 24‑h movement behaviors using CoDa analysis in relation to academic achievement among adolescents older than 12 years. One potential explanation for the inconsistency is the difference in how academic performance was assessed. Among adolescents, whose reading and calculation skills are typically more advanced, movement behaviors may not relate to basic reading and math skills in the same way as they do among children. Consequently, more sophisticated measures of academic performance may be needed to detect potential associations in this age group.

On the other hand, our additional analysis indicated that the time spent in ST relative to other behaviors may be associated differently with reading fluency in boys and girls. ST was positively associated with reading fluency in both boys and girls, although the association was notably weaker among girls. The significant interaction, therefore, suggests that the strength of the association between sedentary behavior and reading fluency varies by gender, being more pronounced among boys. The positive association is consistent with previous studies involving younger participants [30, 32] and is logical, as the content of ST appears to be a critical factor [11]. As noted earlier, supporting reading fluency requires sufficient reading exposure, which entails spending a certain amount of time being sedentary.

Furthermore, theoretical behavior-change scenarios (Fig. 3) were purposely made separately for girls and boys to demonstrate the interaction effect of gender and movement behaviors. Given the finding that girls outperform boys in reading fluency and that the association between ST and reading fluency is weaker among girls, the theoretical behavior‑change scenarios add further nuance to these findings. They suggest that reallocating time from ST to sleep or MPA, as well as from LPA to sleep, appears to widen the gender difference in reading fluency, with potentially more favorable changes among girls and less favorable changes among boys.

A gender-specific association of PA with academic performance has been noticed before among adolescents. Kwak et al. [59] showed that only girls had a positive association between VPA and academic achievement, while Owen et al. [60], in their longitudinal study, found that only girls who increased MVPA over time had greater improvements in academic performance. It is also well established that boys are generally more physically active than girls [35], and girls’ potentially greater need for MPA may be reflected in the pattern observed between movement behaviors and reading fluency in the present study. On the other hand, in some previous studies, the association between PA and academic performance has been an inverted U-shaped curve or even negative [61]. Perhaps in this case, some of the more active boys are spending time in MVPA at the expense of time devoted to reading, studying, and homework, showing a negative association with reading fluency. This observation is supported by a strong divergence in literacy and reading culture between girls and boys. Especially, boys’ leisure reading has decreased, and literacy has declined [62]. Taken together with the interaction results and the theoretical behavior‑change scenarios of this study, and supported by prior evidence, these findings suggest that enhancing reading fluency among adolescents may require different approaches that still acknowledge the need for some ST for both genders: girls may benefit from reallocating time toward sleep and MPA, whereas boys may benefit from maintaining or modestly increasing ST that includes reading exposure.

### Strength and limitations

This study has several strengths. It provides novel insights into the combined association of five movement behaviors (LPA, MPA, VPA, ST, and sleep) with academic performance. Including both children and adolescents enabled examination of the phenomenon across different age groups. Accelerometers’ limitations in measuring cycling and swimming were addressed with diary data. CoDa techniques were used to analyze combined associations. The main limitation is the cross-sectional design, which limits causal inference. Differences in data collection between samples are detailed in the methods section. Academic performance was assessed using different instruments, with only partial reference values, limiting comparability despite within-sample standardization. PA wear-time criteria differed (500 min/day for adolescents, 600 min/day for children), but average wear time varied only slightly (24 min/day). Non-wear gaps (~ 5%) were adjusted by extrapolating observed data. Sleep was assessed by self‑report, which may have introduced measurement error. The samples, inherited from earlier studies, are not fully population-representative: participants were from more educated families, and adolescents had fewer special educational needs. Generalizability is further limited by significant differences between analytic and excluded cases in academic performance, covariates, and 24-h PA behavior.

## Conclusion

The results of this study indicated that lower levels of LPA were linked to better reading fluency among children aged 9–11. Furthermore, the results suggest that the relationship between 24-h movement behavior composition and reading fluency may differ between adolescent boys and girls aged 12–15. Future research is needed to explore gender differences and differences between children and adolescents more closely. From the perspective of 24-h movement behaviors, to support reading fluency, guardians and school staff may need somewhat different strategies for children and adolescents, as well as for girls and boys.

## Supplementary Information

Below is the link to the electronic supplementary material.

Supplementary Material 1.

Supplementary Material 2.

## Data Availability

The data that support the findings of this study are available on request from the corresponding author, [HJS]. The data are not publicly available due to privacy or ethical restrictions.

## Abbreviations

AFIS: Active, fit and smart study
CoDA: Compositional data analysis
CPM: Counts per minute MPA, moderate physical activity
MVPA: Moderate-to-vigorous physical activity
LPA: Light physical activity
PA: Physical activity
ST: Sedentary time
VPA: Vigorous physical activity
