# Supplementary material for: 24-hour movement behaviors in association with academic performance in children and adolescents: cross-sectional compositional data analysis
Source: J Act Sedentary Sleep Behav. 2026 Apr 4;5:8. doi: 10.1186/s44167-026-00099-x (PMC13112822; doi:10.1186/s44167-026-00099-x)
Supplement: Supplementary file 2 — Supplementary Material 2. [file 44167_2026_99_MOESM2_ESM.docx]

**Supplementary material for the article 24-hour movement behaviors in association with academic performance in children and adolescents - cross-sectional compositional data analysis**

**Statistical analyses**

The composition of 24-hour movement behaviors was additionally described by calculating mean ratios for both samples and across the two samples. The full distributions of behaviors were illustrated by calculating and plotting the kernel estimates of empirical density functions of each behavior for both samples.

The relative behavioral profiles according to the level of outcome variables were visualized. It was done by calculating and plotting the relative differences from geometric sample means for three subsamples formed by tertiles (low, middle, high) of values of the outcome variables. Twelve relative behavioral profiles were visualized, representing three tertiles of two outcomes of two samples (3 × 2 × 2 = 12).

**Results**

Supplemental Table S1. Comparison of the analytic sample and the excluded sample.

| **Moving maths sample, children (ages 9‒11)** | **Analytic**  **sample (n=253)** | **Excluded**  **sample (n=144)** | **p-value** |
| --- | --- | --- | --- |
| Categorical variables (%) |  |  |  |
| Gender, boys (%) | 47.0 | 54.2 | 0.210 |
| Special educational needs (%) | **10.3** | **26.4** | **<0.001**** |
| Guardians’ tertiary education (%) | **77.5** | **50.7** | **<0.001**** |
| Continuous variables: M (SD) |  |  |  |
| Age (years) | 9.5 (0.4) | 9.6 (0.6) | 0.329 |
| Body fat (%) | 17.7 (8.5) | 18.6 (8.7) | 0.405 |
| **Academic performance M (SD)** |  |  |  |
| Valid academic performance data | n=253 | n=112 |  |
| Arithmetic fluency (z-score) | 0.0 (1.0) | -0.1 (0.9) | 0.166 |
| Reading fluency (z-score) | **0.1 (1.0)** | **-0.3 (1.1)** | **<0.001**** |
| **Movement behaviors** |  |  |  |
| Valid movement data | n=253 | n=40 |  |
| Sleep (h / d) | 10.1 (0.5) | 10.0 (0.6) | 0.26 |
| ST (h / d) | 7.1 (0.9) | 7.3 (1.0) | 0.146 |
| LPA (h / d) | 4.2 (0.8) | 4.4 (0.7) | 0.19 |
| MPA (min / d) | 68.5 (24.1) | 63.0 (23.0) | 0.136 |
| VPA (min / d) | 23.1 (11.6) | 20.5 (10.5) | 0.074 |
|  |  |  |  |
| **AFIS sample, adolescents (ages 12‒15)** | **Analytic sample (n=174)** | **Excluded sample (n=42)** | **p-value** |
| Categorical variables (%) |  |  |  |
| Gender, boys (%) | 36.8 | 50.0 | 0.162 |
| Special educational needs (%) | 6.9 | 11.9 | 0.446 |
| Guardians’ tertiary education (%) | **72.4** | **54.8** | **0.042*** |
| Continuous variables: M (SD) |  |  |  |
| Age (years) | 13.7 (0.6) | 13.8 (0.6) | 0.259 |
| Body fat (%) | 19.2 (8.1) | 18.5 (8.7) | 0.680 |
| **Academic performance M (SD)** |  |  |  |
| Valid academic performance data | n=174 | n=35 |  |
| Arithmetic fluency (z-score) | 0.1 (1.0) | -0.2 (1.0) | 0.124 |
| Reading fluency (z-score) | 0.0 (1.0) | -0.1 (0.8) | 0.635 |
| **Movement behaviors** |  |  |  |
| Valid movement data | n=174 | n=8 |  |
| Sleep (h / d) | 9.2 (0.7) | 9.4 (0.8) | 0.085 |
| ST (h / d) | 8.9 (1.1) | 9.3 (1.2) | 0.317 |
| LPA (h / d) | **3.0 (0.7)** | **2.6 (0.5)** | **0.018*** |
| MPA (min / d) | **52.8 (26.5)** | **34.3 (18.8)** | **0.006**** |
| VPA (min / d) | 19.7 (11.7) | 15.1 (10.0) | 0.148 |

Supplemental Table S2. Mean ratios of parts of 24-hour PA behavior composition within the full sample and the two age groups.

| **Sample** |  | **Sleep** | **ST** | **LPA** | **MPA** | **VPA** |
| --- | --- | --- | --- | --- | --- | --- |
| Full sample (n=427) | **Sleep** | 1.00 | 1.26 | 2.67 | 10.27 | 31.33 |
|  | **ST** | 0.80 | 1.00 | 2.13 | 8.17 | 24.92 |
|  | **LPA** | 0.37 | 0.47 | 1.00 | 3.84 | 11.71 |
|  | **MPA** | 0.10 | 0.12 | 0.26 | 1.00 | 3.05 |
|  | **VPA** | 0.03 | 0.04 | 0.09 | 0.33 | 1.00 |
| Children n=253) | **Sleep** | 1.00 | 1.44 | 2.41 | 9.39 | 30.08 |
|  | **ST** | 0.69 | 1.00 | 1.67 | 6.52 | 20.90 |
|  | **LPA** | 0.42 | 0.60 | 1.00 | 3.90 | 12.49 |
|  | **MPA** | 0.11 | 0.15 | 0.26 | 1.00 | 3.20 |
|  | **VPA** | 0.03 | 0.05 | 0.08 | 0.31 | 1.00 |
| Adolescents (n=174) | **Sleep** | 1.00 | 1.03 | 3.11 | 11.70 | 33.22 |
|  | **ST** | 0.97 | 1.00 | 3.02 | 11.33 | 32.17 |
|  | **LPA** | 0.32 | 0.33 | 1.00 | 3.76 | 10.67 |
|  | **MPA** | 0.09 | 0.09 | 0.27 | 1.00 | 2.84 |
|  | **VPA** | 0.03 | 0.03 | 0.09 | 0.35 | 1.00 |

Notes: Each cell of the matrix shows the ratio of time spent in two behaviors; behavior on given row to behavior on given column. The three 5x5 matrices separated by horizontal lines represent the combined sample, children’s sample, and adolescents’ sample.

ST, sedentary time, LPA, light physical activity, MPA, moderate physical activity, VPA, vigorous physical activity

Supplemental Table S3. Variation matrix of the behavior composition within the full sample and the two age groups.

| **Sample** |  | **Sleep** | **ST** | **LPA** | **MPA** | **VPA** |
| --- | --- | --- | --- | --- | --- | --- |
| Full sample (n=427) | **Sleep** | 0.00 | 0.05 | 0.06 | 0.20 | 0.35 |
|  | **ST** | 0.05 | 0.00 | 0.17 | 0.32 | 0.45 |
|  | **LPA** | 0.06 | 0.17 | 0.00 | 0.21 | 0.39 |
|  | **MPA** | 0.20 | 0.32 | 0.21 | 0.00 | 0.35 |
|  | **VPA** | 0.35 | 0.45 | 0.39 | 0.35 | 0.00 |
| Children n=253) | **Sleep** | 0.00 | 0.03 | 0.04 | 0.14 | 0.32 |
|  | **ST** | 0.03 | 0.00 | 0.07 | 0.19 | 0.36 |
|  | **LPA** | 0.04 | 0.07 | 0.00 | 0.18 | 0.40 |
|  | **MPA** | 0.14 | 0.19 | 0.18 | 0.00 | 0.29 |
|  | **VPA** | 0.32 | 0.36 | 0.40 | 0.29 | 0.00 |
| Adolescents (n=174) | **Sleep** | 0.00 | 0.02 | 0.07 | 0.26 | 0.40 |
|  | **ST** | 0.02 | 0.00 | 0.11 | 0.34 | 0.47 |
|  | **LPA** | 0.07 | 0.11 | 0.00 | 0.25 | 0.37 |
|  | **MPA** | 0.26 | 0.34 | 0.25 | 0.00 | 0.43 |
|  | **VPA** | 0.40 | 0.47 | 0.37 | 0.43 | 0.00 |

Notes:

ST, sedentary time, LPA, light physical activity, MPA, moderate physical activity, VPA, vigorous physical activity

Supplemental Table S4. Fixed effects of linear mixed models with gender-moderation for arithmetic and reading fluency by age groups.

|  |  | **Children (ages 9‒11), n=253 in 22 groups** | | | | **Adolescents (ages 12‒15), n=174 in 34 groups** | | | |
| --- | --- | --- | --- | --- | --- | --- | --- | --- | --- |
| **Outcome** | **Explanatory variable** | **Beta** | **SE (Beta)** | **95% CI** | **p-value** | **Beta** | **SE (Beta)** | **95% CI** | **p-value** |

| **Arithmetic fluency** |
| --- |

|  | Gender (girl) | 0.28 | 1.28 | -2.16, 2.75 | 0.824 | -0.25 | 1.27 | -2.65, 2.14 | 0.842 |
| --- | --- | --- | --- | --- | --- | --- | --- | --- | --- |
|  | Special educational needs (yes) | -0.90 | 0.20 | -1.28, -0.51 | **<0.001**** | -0.52 | 0.31 | -1.10, 0.06 | 0.092 |
|  | Guardians' education (tertiary education) | 0.25 | 0.14 | -0.02, 0.53 | 0.082 | 0.24 | 0.18 | -0.09, 0.58 | 0.177 |
|  | Age (years) | 0.27 | 0.17 | -0.05, 0.60 | 0.113 | -0.01 | 0.15 | -0.29, 0.26 | 0.936 |
|  | Body fat (%) | -1.61 | 0.79 | -3.18, -0.09 | 0.042 | -1.90 | 1.16 | -4.10, 0.29 | 0.103 |
|  | **First IRL-coordinate of rotated behavior composition** |  |  |  |  |  |  |  |  |
|  | Sleep vs. others | 1.67 | 2.22 | -2.58, 5.96 | 0.454 | -1.40 | 3.18 | -7.41, 4.60 | 0.660 |
|  | Sedentary time vs. others | -1.19 | 1.39 | -3.87, 1.47 | 0.393 | 0.68 | 2.36 | -3.79, 5.15 | 0.775 |
|  | Light PA vs. others | -0.72 | 1.15 | -2.94, 1.47 | 0.530 | 0.72 | 1.29 | -1.72, 3.16 | 0.579 |
|  | Moderate PA vs. others | -0.67 | 0.64 | -1.90, 0.54 | 0.293 | -0.55 | 0.80 | -2.06, 0.96 | 0.490 |
|  | Vigorous PA vs. others | **0.92** | **0.44** | **0.08, 1.76** | **0.037*** | 0.56 | 0.54 | -0.46, 1.57 | 0.300 |
|  | **Interaction terms of gender and first IRL-coordinate** |  |  |  |  |  |  |  |  |
|  | Sleep vs. others | -1.99 | 1.40 | -4.70, 0.68 | 0.156 | 0.15 | 1.84 | -3.33, 3.63 | 0.936 |
|  | Sedentary time vs. others | 1.26 | 0.88 | -0.42, 2.96 | 0.153 | 0.04 | 1.42 | -2.64, 2.71 | 0.980 |
|  | Light PA vs. others | 0.69 | 0.73 | -0.71, 2.10 | 0.346 | -0.36 | 0.75 | -1.78, 1.05 | 0.628 |
|  | Moderate PA vs. others | 0.55 | 0.41 | -0.24, 1.35 | 0.189 | 0.40 | 0.45 | -0.44, 1.25 | 0.370 |
|  | Vigorous PA vs. others | -0.51 | 0.27 | -1.03, 0.01 | 0.062 | -0.22 | 0.31 | -0.81, 0.37 | 0.476 |
|  | Model performance indices | ICC =0.091 | RMSE = 0.843 | R^2^ = 0.166 |  | ICC = 0.000^1^ | RMSE = 0.950 | R^2^ = 0.086 |  |
|  | Explanatory power of behavior composition | Wald = 13.10 | **p = 0.011*** | ΔR^2^ = 0.022 |  | Wald = 1.90 | p = 0.754 | ΔR^2^ = 0.005 |  |

| **Reading fluency** |
| --- |

|  | Gender (girl) | -0.57 | 1.38 | -3.21, 2.10 | 0.679 | -0.07 | 1.22 | -2.38, 2.23 | 0.957 |
| --- | --- | --- | --- | --- | --- | --- | --- | --- | --- |
|  | Special educational needs (yes) | **-0.45** | **0.21** | **-0.86, -0.03** | **0.037*** | -0.59 | 0.30 | -1.15, -0.02 | 0.047 |
|  | Guardians' education (tertiary education) | 0.19 | 0.15 | -0.10, 0.49 | 0.206 | **0.34** | **0.17** | **0.02, 0.66** | **0.048*** |
|  | Age (years) | 0.05 | 0.18 | -0.30, 0.40 | 0.786 | 0.02 | 0.15 | -0.25, 0.30 | 0.879 |
|  | Body fat (%) | -0.17 | 0.84 | -1.83, 1.45 | 0.839 | -1.62 | 1.11 | -3.72, 0.50 | 0.148 |
|  | **First IRL-coordinate of rotated behavior composition** |  |  |  |  |  |  |  |  |
|  | Sleep vs. others | 0.93 | 2.40 | -3.64, 5.56 | 0.699 | **-6.05** | **3.05** | **-11.82, -0.28** | **0.049*** |
|  | Sedentary time vs. others | -0.10 | 1.50 | -2.97, 2.78 | 0.948 | **4.75** | **2.26** | **0.43, 9.03** | **0.038*** |
|  | Light PA vs. others | -0.99 | 1.24 | -3.42, 1.38 | 0.426 | 1.59 | 1.25 | -0.75, 3.97 | 0.202 |
|  | Moderate PA vs. others | -0.35 | 0.69 | -1.67, 0.96 | 0.610 | -0.77 | 0.77 | -2.23, 0.69 | 0.320 |
|  | Vigorous PA vs. others | 0.51 | 0.47 | -0.40, 1.42 | 0.286 | 0.47 | 0.52 | -0.51, 1.45 | 0.361 |
|  | **Interaction terms of gender and first IRL-coordinate** |  |  |  |  |  |  |  |  |
|  | Sleep vs. others | 0.31 | 1.51 | -2.62, 3.19 | 0.837 | 3.37 | 1.77 | 0.03, 6.72 | 0.059 |
|  | Sedentary time vs. others | -0.09 | 0.95 | -1.91, 1.73 | 0.924 | **-2.85** | **1.36** | **-5.42, -0.27** | **0.037*** |
|  | Light PA vs. others | -0.04 | 0.79 | -1.55, 1.51 | 0.964 | -0.74 | 0.72 | -2.13, 0.61 | 0.304 |
|  | Moderate PA vs. others | 0.14 | 0.45 | -0.72, 1.00 | 0.762 | 0.46 | 0.43 | -0.36, 1.28 | 0.288 |
|  | Vigorous PA vs. others | -0.32 | 0.29 | -0.88, 0.24 | 0.274 | -0.23 | 0.30 | -0.80, 0.34 | 0.442 |
|  | Model performance indices | ICC = 0.062 | RMSE = 0.917 | R^2^ = 0.067 |  | ICC = 0.028 | RMSE = 0.894 | R^2^ = 0.145 |  |
|  | Explanatory power of behavior composition | Wald = 1.82 | p = 0.769 | ΔR^2^ = 0.003 |  | Wald = 12.78 | **p = 0.012*** | ΔR^2^ = 0.036 |  |

Notes:

PA, physical activity

* p < 0.05, ** p < 0.01

Interaction terms for gender and the four ratios of the 24‑h behavioral composition are included into the model.

The columns represent the unstandardized regression coefficient (Beta) and its standard error (SE), 95 percent confidence interval (CI), and significance approximated by Satterthwaite's degrees of freedom (p-value).

There is also a single value of intra-class correlation coefficient (ICC), marginal coefficient of determination for fixed effects (R^2^), and root mean squared error (RMSE)

The explanatory power of interaction terms of gender and behavior composition is quantified by Wald's test statistic for comparison of the model with interaction terms and main effects only (Wald), p-value related to Wald test statistic with df=4 (p), and increase of the coefficient of determination for interaction terms (ΔR^2^).

Figure S1. The empirical density function estimates of the parts of 24-hour movement behavior composition. Notes: The distributions of behavior components are calculated by Gaussian kernels with optimized bandwidths (standard deviations) varying between 0.002 and 0.015. The kernel estimates are truncated at zero.

Figure S2. 24-hour movement behavior composition for children and adolescents by arithmetic or reading fluency tertiles. Notes: The Y-axis shows the log-ratios of means of time spent in each behavior within given group compared to the overall average. The higher the deviation is from zero, the more importantly given groups behavior differs from the overall mean composition. In the first row of the panel, the groups are tertiles of the arithmetic fluency of children (A: low, B: middle, C: high tertile). In the second row, the groups are tertiles of the arithmetic fluency of adolescents (D: low, E: middle, F: high tertile). Correspondingly, the third row represents tertiles of reading fluency of children (G: low, H: middle, I: high tertile) and the fourth row for adolescents (J: low, K: middle, L: high tertile).
